# Functional Roles for CD26/DPP4 in Mediating Inflammatory Responses of Pulmonary Vascular Endothelial Cells

**DOI:** 10.3390/cells10123508

**Published:** 2021-12-11

**Authors:** Yukiko Takahashi, Takeshi Kawasaki, Hironori Sato, Yoshinori Hasegawa, Steven M. Dudek, Osamu Ohara, Koichiro Tatsumi, Takuji Suzuki

**Affiliations:** 1 Department of Respirology, Graduate School of Medicine, Chiba University, Chiba 260-8670, Japan; yukkko1000@yahoo.co.jp (Y.T.); tatsumi@faculty.chiba-u.jp (K.T.); suzutaku@chiba-u.jp (T.S.); 2 Department of Applied Genomics, Kazusa DNA Research Institute, Chiba 292-0818, Japan; sim.wis31@gmail.com (H.S.); yhasega@kazusa.or.jp (Y.H.); ohara@kazusa.or.jp (O.O.); 3 Department of Pediatrics, Graduate School of Medicine, Chiba University, Chiba 260-8670, Japan; 4 Division of Pulmonary, Critical Care, Sleep and Allergy, Department of Medicine, University of Illinois at Chicago, Chicago, IL 60612, USA; sdudek@uic.edu

**Keywords:** acute respiratory distress syndrome, lung injury, CD26, DPP4, human lung microvascular endothelial cell, pulmonary endothelial cell, LPS, transcriptome

## Abstract

Excessive inflammation in the lung is a primary cause of acute respiratory distress syndrome (ARDS). CD26/dipeptidyl peptidase-4 (DPP4) is a transmembrane protein that is expressed in various cell types and exerts multiple pleiotropic effects. We recently reported that pharmacological CD26/DPP4 inhibition ameliorated lipopolysaccharide (LPS)-induced lung injury in mice and exerted anti-inflammatory effects on human lung microvascular endothelial cells (HLMVECs), in vitro. However, the mechanistic roles of CD26/DPP4 in lung injury and its effects on HLMVECs remain unclear. In this study, transcriptome analysis, followed by various confirmation experiments using siRNA in cultured HLMVECs, are performed to evaluate the role of CD26/DPP4 in response to the pro-inflammatory involved in inflammation, barrier function, and regenerative processes in HLMVECs after pro-inflammatory stimulation. These are all functions that are closely related to the pathophysiology and repair process of lung injury. Confirmatory experiments using flow cytometry; enzyme-linked immunosorbent assay; quantitative polymerase chain reaction; dextran permeability assay; WST-8 assay; wound healing assay; and tube formation assay, reveal that the reduction of CD26/DPP4 via siRNA is associated with altered parameters of inflammation, barrier function, and the regenerative processes in HLMVECs. Thus, CD26/DPP4 can play a pathological role in mediating injury in pulmonary endothelial cells. CD26/DPP4 inhibition can be a new therapeutic strategy for inflammatory lung diseases, involving pulmonary vascular damage.

## 1. Introduction

Acute respiratory distress syndrome (ARDS) is characterized by enhanced pulmonary vascular permeability leading to a non-cardiac pulmonary edema. Excessive inflammation in the lungs is a major trigger for ARDS. The pathophysiology of ARDS includes inflammation; the uncontrolled activation of leukocytes, platelets, and coagulation systems; and increased permeability of the endothelial and alveolar epithelial barriers [1]. Pulmonary vascular endothelial cells and lung epithelial cells generate pro-inflammatory signals during ARDS progression [2]. The pathological phases of ARDS are classified into three stages: exudative, proliferative, and fibrotic. The exudative stage is characterized by increased pulmonary vascular permeability and increased neutrophils in the alveolar septum and airspaces, along with the death of epithelial and endothelial cells. The proliferative stage is characterized by fibroblast proliferation and type 2 pneumocyte hyperplasia [3]. This phase is also important in terms of endothelial repair and regeneration, which are essential steps for recovering from ARDS [4]. Although numerous studies have focused on the mechanisms and treatment strategies of ARDS, no effective drug therapies have been established.

CD26/dipeptidyl peptidase-4 (DPP4) is a transmembrane protein expressed in a variety of cells that also exists as a soluble protein in tissue and the circulation. CD26/DPP4 exerts its peptidase activity towards various proteins, such as incretin hormones, and CD26/DPP4 inhibitors have been developed as therapeutic agents for diabetes. In addition, CD26/DPP4 can participate in immune stimulation and the promotion of inflammation [5,6]. Previous studies showed that pharmacological CD26/DPP4 inhibition has cardiovascular protective and anti-inflammatory effects in the vessels [7].

CD26/DPP4 is widely expressed in a variety of cell types in lung tissue, such as type I and II alveolar cells, alveolar macrophages, and vascular endothelia [8,9,10], and has been recently suggested to be a therapeutic target in lung diseases [11]. For example, previous reports have indicated that CD26/DPP4 inhibitors have a protective effect on lung ischemia-reperfusion injury through promoting the recruitment of endothelial progenitor cells by retaining SDF-1/CXCL12 activity [12,13]. Another study suggested that CD26/DPP4 inhibitors can be novel prophylactic drugs for chronic allograft dysfunction after clinical transplantation [14]. Furthermore, recent studies have reported that CD26/DPP4 contributed to non-typeable H. influenzae-induced lung inflammation in COPD [15], and can participate in the pathogenesis of pulmonary hypertension [16]. We previously demonstrated that CD26/DPP4 inhibition by sitagliptin ameliorated lipopolysaccharide (LPS)-induced lung injury in mice through its anti-inflammatory effects on pulmonary endothelial cells, indicating a relationship between pulmonary endothelium in ARDS and CD26/DPP4 [17]. However, the mechanistic roles of CD26/DPP4 in the pulmonary endothelium at each stage of ARDS remain unclear.

Studies of pulmonary vascular endothelial cells are essential for understanding the mechanism of ARDS because these cells exert multiple relevant effects, such as the generation of inflammatory mediators and the regulation of intracellular adhesion, which are related to the endothelial barrier [2]. Pulmonary endothelial injury as a key disease mechanism and the contribution of progenitor cells in mediating endothelial repair have also been suggested as important processes in lung injury [18]. We recently reported that CD26/DPP4 inhibition has anti-inflammatory effects on human lung microvascular endothelial cells (HLMVECs) [17], indicating that CD26/DPP4 is involved in lung endothelial cell functions related to the pathophysiology of ARDS.

In this study, we examined the roles of CD26/DPP4 in ARDS pathology, focusing on pulmonary vascular endothelial cells. We performed an in vitro transcriptome analysis and functional experiments employing specific *DPP4* knockdown using microRNA on HLMVECs, to evaluate the response of these cells to the pro-inflammatory stimulus LPS.

## 2. Materials and Methods

### 2.1. Human Lung Endothelial Cell Culture

HLMVECs were obtained from Lonza (Basel, Switzerland) and cultured in endothelial growth medium-2 supplemented with 10% fetal bovine serum. The cells were incubated at 37 °C in a 5% CO_2_ incubator and used at passages 6–8 for all experiments.

### 2.2. Reagents

Non-specific control siRNA (siCon) (Cat# 4390843: Silencer™ Select Negative Control No. 1 siRNA) and *DPP4* siRNA (Cat# 4392421: siRNA ID s4255) were purchased from Thermo Fisher Scientific (Waltham, MA, USA). *Escherichia coli* LPS (O127:B8, L3137) and all other reagents were purchased from Merck (Darmstadt, Germany), unless otherwise specified.

### 2.3. Transfections with Silencing RNA

For siRNA transfection, the Lipofectamine™ RNAiMAX Transfection Reagent (Invitrogen, Carlsbad, CA, USA) was used. The cells were transfected with siRNA at a 60% confluence, according to the manufacturer’s protocol, and then challenged with LPS at 72 h after siRNA treatment. The selective silencing of CD26/DPP4 was confirmed using flow cytometry analysis and quantitative PCR.

### 2.4. RNA Sequencing

Four primary cell lines of HLMVECs were used; the donors included a 44-year-old Hispanic male, 38-year-old Caucasian male, 59-year-old Hispanic female, and 30-year-old Hispanic female, according to the information from Lonza. Total RNA was isolated from the HLMVECs and stored in Isogen (Nippon Gene, Tokyo, Japan). Two milliliters of this solution was vigorously vortexed and then centrifuged after adding 400 μL of chloroform. The supernatants were removed, and 40 μg of glycogen (Roche, Basel, Switzerland) was added. RNA was precipitated by adding 1000 μL of isopropyl alcohol. The solution was vortexed vigorously and centrifuged. The RNA pellets were washed with 75% ethanol and then dissolved in 10 μL RNase-free water. The concentration and quality of the RNA were verified using an Agilent 2100 Bioanalyzer (Agilent Technologies, Santa Clara, CA, USA).

Purified total RNA (500 ng) with an RIN value of >9 was used for RNA library preparation, according to the instructions of the QuantSeq 3′mRNA-Seq Library Prep Kit FWD for Illumina (Lexogen, Vienna, Austria). The libraries were amplified using 12 cycles of polymerase chain reaction (PCR). The RNA libraries were sequenced using an Illumina NextSeq 500 system (75 cycles; San Diego, CA, USA). FASTQ files were prepared with reads using bcl2fastq ver2.17 (Illumina). The quality of the FASTQ sequence data was assessed using FastQC v0.11.9 (Illumina). After removing the adapter sequences from the raw reads, trimmed reads were aligned using STAR v2.7.6a to the GRCh38 human reference genome. Reads per million values were calculated using samtools v1.11 and htseq-count v0.12.4.

### 2.5. Differentially Expressed Genes (DEGs), Gene Ontology (GO), and the Kyoto Encyclopedia of Genes and Genomes (KEGG) Pathway Enrichment Analysis

The expression levels of the genes identified in the transcriptome were normalized and compared. Hierarchical clustering and heat maps were created using the Qlucore omics explorer software program (Qlucore AB, Lund, Sweden). Differentially expressed genes (DEGs) were detected for each gene between the two type samples. The fold-changes between each group were >1.5 (upregulated) or <0.66 (downregulated) (*p* < 0.1). Significantly, over-represented functional categories were identified using Enricher [12]. Genes significantly upregulated or downregulated in “siRNA for *DPP4* (siRNA) and PBS vs. negative control of siRNA (NC) and PBS”, “NC/PBS vs. NC/LPS”, or “NC/LPS vs. siRNA/LPS” were annotated. Subsequently, the gene ontology (GO) terms and the Kyoto Encyclopedia of Genes and Genomes (KEGG) pathways were identified. Selected GO terms and KEGG pathways were considered as significant at *p* < 0.05.

### 2.6. Flow Cytometry Analysis

Cultured HLMVECs were detached from culture plates using STEMPRO ACCUTASE (Thermo Fisher Scientific), pretreated with Human TruStain FcX (BioLegend, San Diego, CA, USA) to block Fc receptors for 10 min, followed by incubation with anti-human CD26-phycoerythrin (BioLegend) or anti-human ICAM-1-phycoerythrin (BioLegend) in the dark at 4 °C for 20 min. Cell fluorescence was measured using a BD FACSCanto™ II (BD Biosciences, Franklin Lakes, NJ, USA), and the data were analyzed using FlowJo software (TreeStar, Ashland, OR, USA).

### 2.7. Real-Time Quantitative PCR

Total RNA from HLMVECs was extracted using TRIzol and a Direct-zol RNA MiniPrep Plus kit (Zymo Research Corporation, Irvine, CA, USA). The RNA was reverse-transcribed via PCR with SuperScript IV VILO Master Mix (Thermo Fisher Scientific) to synthesize single-stranded cDNA. The cDNA samples were amplified via quantitative PCR (qPCR) with Taqman Fast Universal PCR Master Mix (Thermo Fisher Scientific), using the GeneAmp PCR System (Thermo Fisher Scientific). Specific primers were designed using web software from the Universal ProbeLibrary Assay Design Center (Roche Applied Science, Waltham, MA, USA). The expression level of each target gene was normalized to the HPRT1 threshold cycle (CT) values and calculated using the 2^−ΔΔCt^ method. ΔΔCT =  (target gene CT of experimental group − reference gene CT of experimental group) − (target gene CT of control group − reference gene CT of control group).

### 2.8. Enzyme-Linked Immunosorbent Assay (ELISA) of Conditioned Medium

Cultured HLMVECs were treated with an siRNA or vehicle (water) for 72 h, followed by treatment with LPS (1 µg/mL) or vehicle (PBS) for 6 h. The harvested culture medium was centrifuged (500× *g* for 10 min at 4 °C), and the supernatant was collected for ELISA. Human interleukin (IL)-6 and IL-8 levels were measured using ELISA kits (BioLegend), according to the manufacturer’s protocols.

### 2.9. Cell Proliferation Assay

A cell proliferation assay was performed to assess the number of viable cells using Cell Counting Kit-8 (WST-8) (Dojindo Molecular Technologies, Kumamoto, Japan), according to the manufacturer’s protocol. HLMVECs (5000 cells/well) treated with siRNA or vehicle for 72 h were detached using ACCUTASE and precultured on a 96-well plate for 24 h, followed by treatment with LPS (1 µg/mL) or PBS (-) for 18 h. The cells were cultured with 10 μL WST-8 in each well at 37 °C for 2 h. Cell viability was measured as the absorbance (optical density (OD)) read at 450 nm using a microplate reader. The result was calculated using the following formula: cell viability  = (treatment group OD − blank group OD)/(control group OD − blank group OD).

### 2.10. Wound Healing Assay

A wound was established by manually scraping the confluent cell monolayer using a 200 µL pipette tip. Initial images of the culture plates were acquired as the reference points. After incubating the plates for 7 h at 37 °C in a 5% CO_2_ incubator, a second image was acquired. The wounded region lacking cells was measured using ImageJ software (National Institutes of Health, Bethesda, MD, USA).

### 2.11. Tube Formation Assay

Matrigel (Corning, Inc., Corning, NY, USA) was thawed at 4 °C, and then 96-well plates were coated with 50 µL/well of Matrigel. The cells were detached from culture plates using ACCUTASE, and the volume was adjusted with a complete medium to obtain 2 × 10^4^/200 µL, and the cell mixture (200 µL) was added to each Matrigel-coated well. The plate was incubated for 6 h at 37 °C in a 5% CO_2_ incubator. Briefly, for the quantification of the tube formation, the number of branch nodes was counted and the lengths of all the tubes were measured per one field of a microscope image of each well, as shown in Supplementary Figure S1 [19].

### 2.12. In Vitro Vascular Permeability Assay

Endothelial cell monolayer permeability was assessed using an in vitro vascular permeability assay kit (Millipore, Billerica, MA, USA). The inserts were coated with collagen and included a membrane with 1 µm pores. HLMVECs were treated with siRNA for 3 days and then cultured in the inserts on a 24-well plate to form an endothelial cell monolayer over the membrane (3 × 10^5^ cells per insert). The cells were incubated for another 2 days, followed by LPS challenge (1 µg/mL) for 4 h. Vascular permeability was quantified using fluorescein isothiocyanate (FITC)-dextran. FITC-dextran diluted by 1:40 with complete culture medium was added to the inserts (150 µL each), and the plate was incubated at room temperature for 20 min. The reaction was stopped, and the fluorescence signal was detected using a Qubit 4 fluorometer (Thermo Fisher Scientific) at 485 nm excitation.

### 2.13. Statistical Analysis

The results are expressed as the mean ± standard deviation (SD). One-way analysis of variance was used for multiple-group comparisons, followed by Tukey’s post hoc test. Student’s t-test was used to compare two groups. Statistical analyses were performed using GraphPad Prism 6 software (GraphPad, Inc., La Jolla, CA, USA). The statistical significance was set at *p* < 0.05.

## 3. Results

### 3.1. CD26/DPP4 Expression Levels in HLMVECs Treated with siRNA Were Decreased

**Figure 1:**
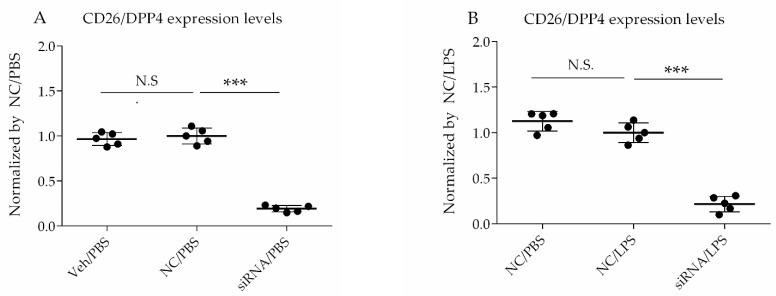

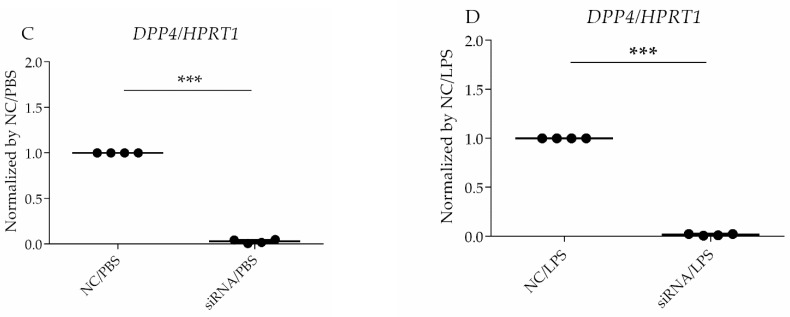
Validation of CD26/DPP4 expression levels in HLMVECs treated with siRNA. HLMVECs were transfected with non-specific (NC) or *DPP4*-specific siRNA for 72 h, and then challenged with LPS (1 µg/mL) or PBS for 18 h. (**A**) CD26/DPP4 expression levels in cultured HLMVECs after vehicle or siRNA treatment followed by PBS challenge (measured by mean fluorescent intensity (MFI) using flow cytometry analysis); (**B**) CD26/DPP4 expression levels in cultured HLMVECs after siRNA treatment followed by LPS or PBS challenge (measured by MFI using flow cytometry analysis); (**C**) mRNA expression levels of *DPP4* relative to those of the control mRNA *HPRT1* in HLMVECs after siRNA treatment followed by PBS treatment as the baseline condition (measured by quantitative PCR); and (**D**) mRNA expression levels of DPP4 relative to those of the control mRNA *HPRT1* in HLMVECs after siRNA treatment followed by LPS treatment (measured by quantitative PCR). *** *p* < 0.01. Values are means ± SD of three independent experiments. N.S., Not significant; Veh., Vehicle; and NC, Non-specific control RNA.

In this study, siRNA was used to specifically decrease CD26/DPP4 expression to assess its specific functions in HLMVECs. CD26/DPP4 expression was suppressed in cultured HLMVECs by incubation with siRNA for 72 h, and then PBS or LPS was added to the cells and cultured for another 18 h. To confirm whether the siRNAs sufficiently suppressed CD26/DPP4 expression in HLMVECs, both protein and gene levels of CD26/DPP4 were measured. Treatment with siRNA for *DPP4* significantly reduced CD26/DPP4 protein levels by ~70% compared to in the non-specific control group (*p* < 0.05) (Figure 1A), and the reduction in protein levels remained unchanged at 18 h after LPS challenge (Figure 1B). Reduced *DPP4* mRNA expression in cultured HLMVECs was also confirmed using real time qPCR under baseline conditions without inflammatory stimulation (Figure 1C) and inflammatory conditions by LPS stimulation (Figure 1D).

### 3.2. Transcriptome Analysis and Verification Experiments: Identification of Effects of DPP4 Knockdown on HLMVECs

Transcriptome analysis of cultured cells is useful for measuring the expression of a wide variety of mRNAs to comprehensively reflect the expression status of genes in cells. We first performed transcriptomic analysis for HLMVECs treated with/without *DPP4* siRNA, with or without LPS stimulation, and then conducted confirmation experiments. The expression levels of the representative factors known to be involved in the pathology of ARDS are summarized in Table 1. *DPP4* and *ICAM1* were downregulated by *DPP4* siRNA, whereas *TNF*, *IL6*, and *CXCL8* remained unchanged.

#### 3.2.1. Transcriptome Analysis of the Effects of *DPP4* Knockdown on HLMVECs without LPS Stimulation

**Figure 2:**
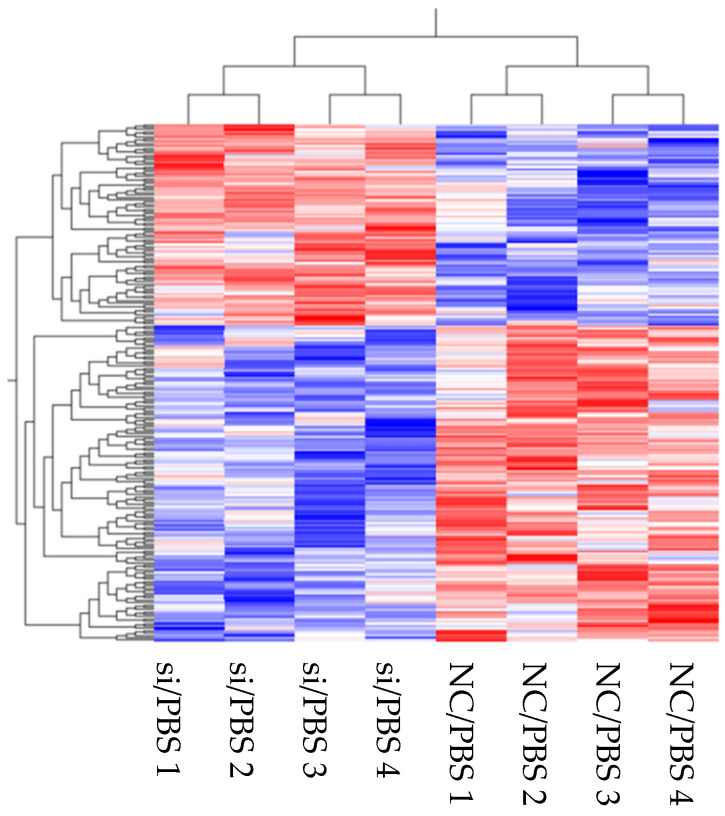
Transcriptomic analysis of HLMVECs under baseline conditions without LPS challenge. Heatmap represents transcriptomic analysis of differentially expressed genes between the *DPP4* siRNA (NC)—and negative control siRNA (NC)—treated HLMVECs.

We first evaluated the effects of *DPP4* knockdown on HLMVECs under baseline conditions without inflammatory stimulation, to identify molecular alterations at the transcriptional level (HLMVECs without LPS stimulation, hereinafter, referred to as “baseline condition”). We identified genes that were upregulated or downregulated in HLMVECs treated with *DPP4* siRNA compared to control siRNA. Hierarchical clustering analysis revealed the trends in DEGs between HLMVECs treated with *DPP4* siRNA and negative control siRNA (see heatmap Figure 2). Following *DPP4* knockdown, 384 genes were upregulated in HLMVECs, whereas 501 genes were downregulated. Enrichment analysis (GO and KEGG pathways) suggested that a reduction in CD26/DPP4 affects inflammation, as indicated by terms such as MAPK and TNF signaling pathways, as well as proliferation and angiogenesis, as indicated by terms such as positive regulation of endothelial cell proliferation (GO:0001938) and angiogenesis involved in wound healing (GO:0060055) (Table 2A,B).

#### 3.2.2. Verification Experiments of the Effects of *DPP4* Knockdown on HLMVECs without LPS Stimulation

Transcriptomic analysis indicated that *DPP4* knockdown affected HLMVEC functions related to ARDS pathophysiology, such as inflammation and regenerative processes. Therefore, we confirmed the effects of *DPP4* knockdown through validation experiments.

#### 3.2.3. Assessment of the Pro-Inflammatory Parameter Intercellular Adhesion Molecule 1 (ICAM-1)

**Figure 3:**
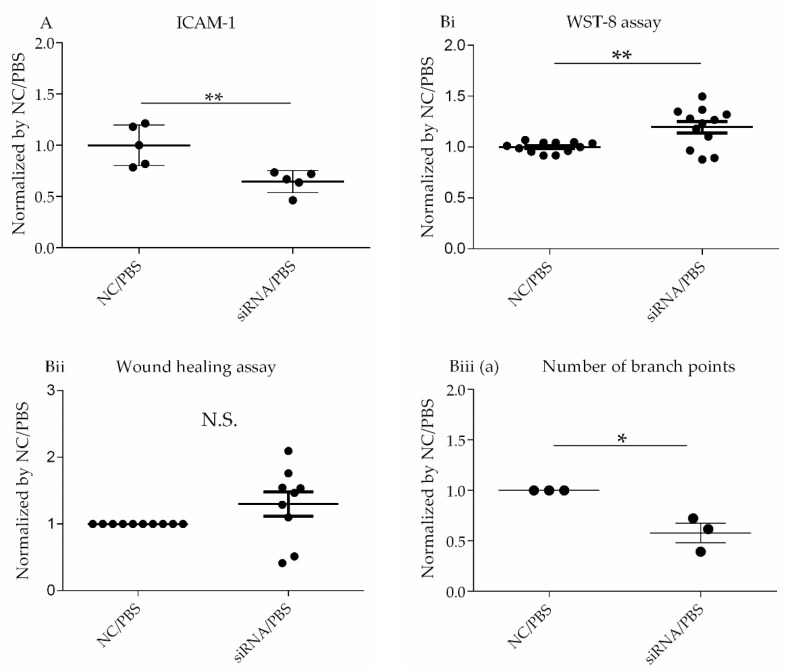

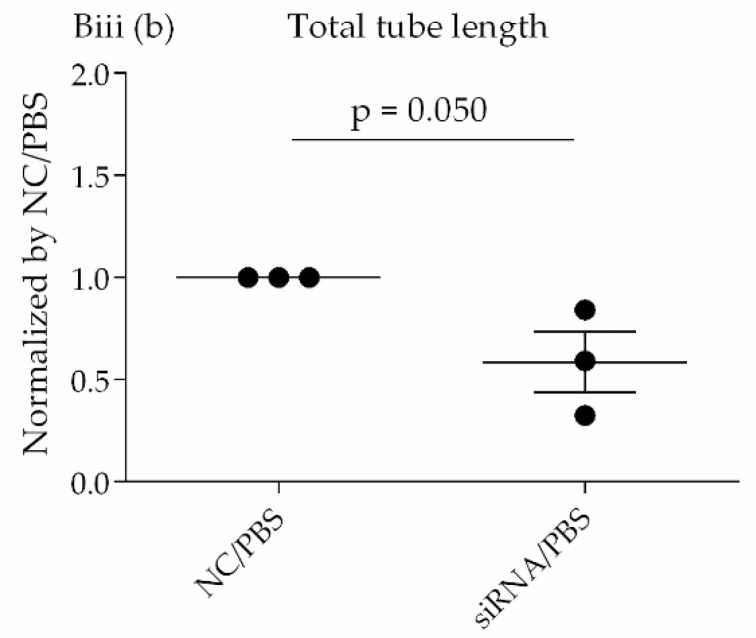
Confirmation studies of HLMVECs under baseline conditions. (**A**) Assessment of ICAM-1 expression level on HLMVECs. HLMVECs were transfected with non-specific or *DPP4*-specific siRNA for 72 h, and then challenged with PBS for 18 h. ICAM-1 expression on HLMVECs was measured by mean fluorescence intensity (MFI) using flow cytometry analysis. ICAM-1 levels in cultured HLMVECs after siRNA treatment followed by PBS challenge are shown. * *p* < 0.05, ** *p* < 0.01. The values are means ± SD of three or more independent experiments. N.S., Not significant; NC, Non-specific control; siRNA, *DPP4* siRNA. (**B**) Assessment of regenerative process under baseline conditions. (**i**) WST-8 assay to evaluate proliferative capacity of HLMVECs. HLMVECs were transfected with non-specific or *DPP4*-specific siRNA for 72 h, and then challenged with PBS for 18 h. Proliferation was assessed using a WST-8 assay. (**ii**) Wound healing assay to evaluate migratory ability of HLMVECs. HLMVECs were transfected with non-specific or *DPP4*-specific siRNA for 72 h, and then challenged with PBS for 18 h. HLMVEC migration was measured by wound healing assay. (**iii**) Tube formation assay to evaluate the lumen forming ability of HLMVECs. The lumen forming ability of HLMVECs was evaluated by tube formation assay. HLMVECs were transfected with non-specific or *DPP4*-specific siRNA for 72 h, and then cultured in Matrigel-coated wells, followed by PBS for 6 h. The number (**Biii (a)**) and lengths of tubes (**Biii (b)**) formed by the cells were counted by microscopy. * *p* < 0.05, ** *p* < 0.01. The values are presented as the mean ± SD of three or more independent experiments. N.S., not significant; NC, non-specific control; siRNA, *DPP4* siRNA.

ICAM-1, also known as CD54, is a transmembrane glycoprotein and a key molecule involved in inflammatory processes. This protein functions as a ligand for the leukocyte adhesion protein lymphocyte function-associated antigen 1 and is upregulated by LPS, leading to neutrophil transmigration [20]. The increased expression of the adhesion molecules is an important step in the inflammatory response and is one of the hallmarks of lung injury; therefore, we evaluated whether the reduction of CD26/DPP4 levels using siRNA affects the expression of ICAM-1 in HLMVECs. HLMVECs were transfected with non-specific or *DPP4*-specific siRNA for 72 h, and then challenged with PBS for 18 h. ICAM-1 expression in HLMVECs was measured as the mean fluorescence intensity (MFI) using flow cytometry analysis. ICAM-1 levels in these cells were significantly decreased by *DPP4* siRNA compared to non-specific siRNA treatment (Figure 3A).

#### 3.2.4. Assessment of the Regenerative Process

Endothelial cell proliferation is an important aspect of endothelial barrier repair [21,22,23,24]. Therefore, we evaluated the proliferation capacity of HLMVECs using a WST-8 assay. In HLMVECs treated with *DPP4* siRNA, the proliferative capacity was significantly increased compared to that of cells treated with negative control siRNA (Figure 3Bi).

The migratory ability of ECs is also related to endothelial regeneration [25]. HLMVEC migration was evaluated in a wound healing assay by measuring the wound closure ratio compared to that of negative control cells. In this assay, there was no significant difference between cells treated with *DPP4* siRNA and those treated with negative control siRNA (Figure 3Bii).

To assess another aspect of endothelial repair, the lumen-forming ability of HLMVECs was evaluated by performing a tube formation assay. After 6 h of seeding the cells on Matrigel-coated wells, the tube formation was quantified by measuring the number and lengths of tubes in the microscopic images of each well. Both the number and lengths of tubes trended toward a reduced level in cells treated with *DPP4* siRNA compared to those in negative control samples (Figure 3Biii(a,b).

### 3.3. Transcriptome Analysis and Confirmation Experiments: Effects of DPP4 Knockdown on HLMVECs after LPS Stimulation

#### 3.3.1. Transcriptome Analysis of the Effects of LPS Stimulation on HLMVECs

**Figure 4:**
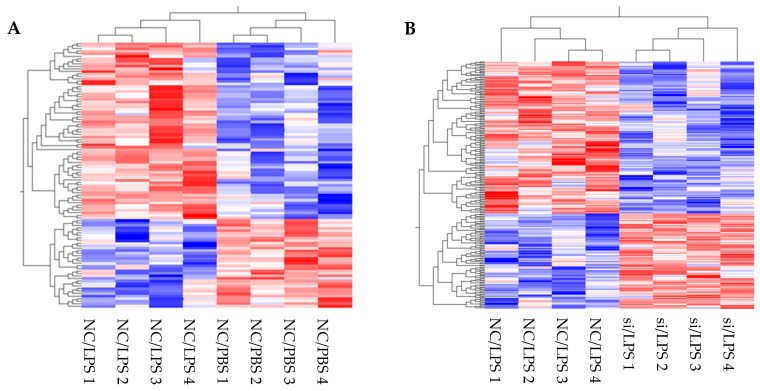
Transcriptomic analysis of HLMVECs with LPS challenge. Heatmap represents the transcriptomic analysis of differentially expressed genes in HLMVECs after LPS stimulation. (**A**): Negative control siRNA (NC)/LPS vs. NC/PBS, and (**B**): *DPP4* siRNA (siRNA)/LPS vs NC/LPS.

The effects of LPS on HLMVECs at the transcriptional level are complex and unclear. Therefore, we first evaluated these effects to identify whole-molecule alterations. Hierarchical clustering analysis revealed the trends in DEGs between LPS- and PBS-treated HLMVECs (see heatmap Figure 4A). Following LPS stimulation, 496 genes were upregulated in HLMVECs, whereas 403 genes were downregulated. LPS is a known ligand of TLR4 and, therefore, TLR4 signal-related genes have also been studied [26,27]. Enrichment analysis indicated that LPS upregulates the TLR4 signaling pathway and other proinflammatory pathways, such as TNF signaling, NF-kappa B signaling, chemokine signaling, PI3K-Akt signaling, and MAPK signaling. It also downregulates tight junctions (Table 3). The expression levels of representative factors involved in ARDS pathophysiology are summarized in Table 4. *DPP4* and *TNF* were unchanged after LPS challenge, whereas *ICAM1*, *IL6*, and *CXCL8* were upregulated.

#### 3.3.2. Transcriptome Analysis of the Effects of *DPP4* Knockdown on HLMVECs after LPS Stimulation

We next analyzed the effects of *DPP4* knockdown using siRNA on HLMVECs stimulated by LPS to identify molecular alterations at the transcriptional level. Transcriptome analysis revealed DEGs between HLMVECs treated with *DPP4* siRNA and negative control siRNA (see heatmap Figure 4B). Following *DPP4* siRNA treatment, 354 genes were upregulated in HLMVECs, and 463 genes were downregulated. Enrichment analysis suggested that reduction of CD26/DPP4 altered LPS-induced inflammation, TNF signaling pathway, proliferation, and angiogenesis. These effects were indicated by terms such as positive regulation of endothelial cell proliferation (GO:0001938), regulation of endothelial cell proliferation (GO:0001936), endothelial cell proliferation (GO:0001935), and positive regulation of vasculature development (GO:1904018). Effects on monolayer permeability were also suggested, as indicated by the terms actin filament bundle assembly (GO:0051017), actin filament bundle organization (GO:0061572), and focal adhesion (Table 5). The expression levels of the representative factors involved in ARDS pathophysiology are summarized in Table 6. Upon *DPP4* siRNA treatment, *DPP4* was downregulated by *DPP4* siRNA, *ICAM 1* trended toward downregulation (*p* = 0.17, n = 4), and *TNF*, *IL6*, and *CXCL8* expressions were unchanged.

#### 3.3.3. Confirmation of the Effects of *DPP4* Knockdown on HLMVECs after LPS Stimulation

Transcriptomic analysis indicated that *DPP4* knockdown affects the functions of HLMVECs under baseline conditions in terms of parameters relevant to ARDS, such as inflammation, permeability, and the regenerative process. Therefore, we next evaluated the effects of *DPP4* knockdown on these parameters in HLMVECs following LPS stimulation.

#### 3.3.4. Assessment of Pro-Inflammatory Parameters

**Figure 5:**
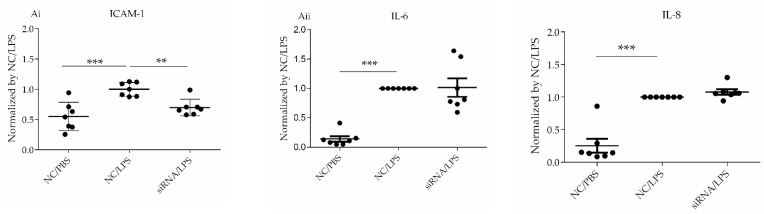

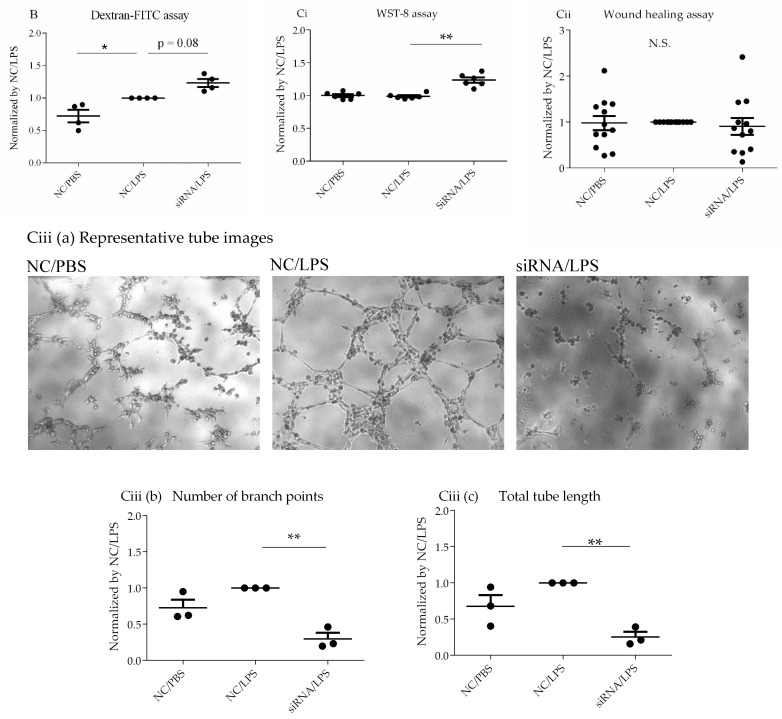
Confirmation studies of HLMVECs after LPS challenge. (**A**) Assessment of pro-inflammatory parameters. (**i**) ICAM-1 expression levels on HLMVECs. HLMVECs were transfected with non-specific or *DPP4*-specific siRNA for 72 h, and then challenged with LPS (1 µg/mL) or PBS for 18 h. ICAM-1 expression on HLMVECs was measured by mean fluorescence intensity (MFI) using flow cytometry analysis. (**ii**) Release of pro-inflammatory cytokines IL-6 and IL-8. HLMVECs were transfected with non-specific or *DPP4*-specific siRNA for 72 h, and then challenged with LPS (1 µg/mL) or PBS for 6 h. Secreted cytokine levels in the conditioned media were measured by ELISA, and the values were normalized to that of non-specific siRNA/LPS-treated groups. Levels of both IL-6 and IL-8 were increased by LPS challenge, but no differences were observed between the groups treated with non-specific RNA and *DPP4* siRNA. * *p* < 0.05, ** *p* < 0.01, *** *p* < 0.001. Values are means ± SD of three or more independent experiments. N.S., Not significant; NC, Non-specific control; siRNA, *DPP4* siRNA. (**B**) Assessment of monolayer permeability in HLMVECs. Permeability was evaluated by dextran-FITC assay. LPS enhanced monolayer permeability of the HLMVECs. *DPP4* siRNA further increased HLMVEC permeability after LPS stimulation. (**C**) Assessment of regenerative process in HLMVECs after LPS. (**i**) WST-8 assay to evaluate proliferative capacity of HLMVECs. HLMVECs were transfected with non-specific, or *DPP4*-specific siRNA for 72 h, and then challenged with LPS (1 µg/mL) or PBS for 18 h. Proliferation was assessed using a WST-8 assay. (**ii**) Wound healing assay to migratory ability of HLMVECs. HLMVECs were transfected with non-specific, or *DPP4*-specific siRNA for 72 h, and then challenged with LPS (1 µg/mL) for 18 h. The migratory ability of HLMVECs was measured by wound healing assay. (**iii**) Tube formation assay to evaluate lumen forming ability of HLMVECs. The lumen forming ability of HLMVECs was evaluated by tube formation assay. HLMVECs, which were transfected with non-specific or *DPP4*-specific siRNA for 72 h, and were cultured in Matrigel-coated wells, followed by LPS challenge LPS (1 µg/mL) or PBS for 6 h. The number and lengths of tubes formed by the cells were counted using microscopy. The representative tube images (**a**), the branch number of tubes (**b**), and the lengths of tubes (**c**) are shown. * *p* < 0.05, ** *p* < 0.01, *** *p* < 0.001. The values are means ± SD of three or more independent experiments. N.S., Not significant; NC, Non-specific control; siRNA, *DPP4* siRNA.

HLMVECs were transfected with non-specific, or two types of *DPP4*-specific siRNA for 72 h, and then challenged with LPS (1 µg/mL) or PBS for 18 h. ICAM-1 expression in HLMVECs was measured as the MFI using flow cytometry analysis. The levels of ICAM-1 were significantly increased by LPS challenge. *DPP4* siRNA significantly attenuated this LPS-induced increase in ICAM-1 expression by ~30% compared with non-specific siRNA treatment (*p* < 0.05) (Figure 5Ai).

IL-6 and IL-8 are pro-inflammatory cytokines produced by many cell types, including HLMVECs. IL-6 is a biomarker of lethal sepsis, and IL-8 is a neutrophil chemotactic factor, and both cytokines play key roles in lung injury [28,29]. To examine whether suppression of CD26/DPP4 affects these parameters of inflammation in HLMVECs after LPS stimulation, the conditioned media were collected and examined using ELISA to quantify the release of the pro-inflammatory cytokines IL-6 and IL-8. HLMVECs were transfected with *DPP4*-specific siRNA for 72 h and then challenged with LPS (1 µg/mL) or PBS for 6 h. Although the concentrations of both IL-6 and IL-8 were significantly increased by LPS challenge, no differences were observed between the groups treated with non-specific RNA and *DPP4* siRNA (Figure 5Aii).

#### 3.3.5. Endothelial Permeability

Endothelial permeability was evaluated using a dextran-FITC assay. Under these conditions, LPS trended toward the enhanced the permeability of the HLMVEC monolayer. *DPP4* siRNA further increased HLMVEC permeability after LPS stimulation (Figure 5B).

#### 3.3.6. Assessment of the Regenerative Process

LPS stimulation did not significantly alter the proliferative capacity of HLMVECs treated with control siRNA. However, in HLMVECs treated with *DPP4* siRNA, the proliferative capacity was significantly increased after LPS stimulation compared with that in cells treated with negative control siRNA (Figure 5Ci).

The migratory ability of HLMVECs was evaluated in a wound healing assay by measuring the wound closure ratio compared to that of negative control cells. HLMVEC migration was not significantly altered by LPS challenge. Moreover, there was no significant difference between cells treated with *DPP4* siRNA and those treated with negative control siRNA under LPS stimulation (Figure 5Cii and Figure S2).

The lumen-forming ability of HLMVECs was evaluated using a tube formation assay. At 6 h, after adding the cells to Matrigel-coated wells, the tube formation was quantified by measuring the number and lengths of the tubes in the microscopic images of each well. LPS stimulation did not significantly alter the tube formation in HLMVECs treated with control siRNA. However, both the number and lengths of the tubes significantly decreased in cells treated with *DPP4* siRNA compared to those in the negative control under LPS stimulation (Figure 5Ciii).

## 4. Discussion

We previously reported that CD26/DPP4 inhibition by sitagliptin attenuates LPS-induced lung injury in mice and has anti-inflammatory effects on LPS-stimulated HLMVECs [17]. In the present study, we substantially advance this prior work by performing in vitro experiments using *DPP4* siRNA to better understand the detailed mechanisms by which CD26/DPP4 expression is related to the functions of HLMVECs under baseline and inflammatory conditions. We employed transcriptome analysis to detect DEGs and identify the possible functions affected by *DPP4* knockdown, followed by confirmatory functional experiments in HLMVECs. These results suggest that CD26/DPP4 expression is related to multiple important functions in HLMVECs, including inflammation, barrier function, and regenerative processes, all of which are all known as hallmarks of ARDS pathophysiology and essential processes for recovery from lung injury.

First, transcriptome analysis of enriched DEGs suggested changes in multiple cell functions and pathways following *DPP4* knockdown or LPS stimulation. Under baseline conditions without LPS stimulation, enrichment analysis (GO and KEGG pathways) suggested that CD26/DPP4 reduction affects inflammation, as indicated by terms such as MAPK or TNF signaling pathways (Table 2B), as well as proliferation and angiogenesis, as indicated by terms such as positive regulation of endothelial cell proliferation (GO:0001938) and angiogenesis involved in wound healing (GO:0060055) (Table 2A,B). Under the inflammatory state induced by LPS, enrichment analysis suggested that upregulation occurs in the TLR4 signaling pathway and other pro-inflammatory pathways, such as TNF signaling, NF-kappa B signaling, chemokine signaling, PI3K-Akt signaling, and MAPK signaling (Table 3). LPS also downregulates tight junctions, which can lead to increased permeability (Table 3). In addition, enrichment analysis suggested that a reduction of CD26/DPP4 expression via siRNA alters LPS-induced inflammation, as indicated by the terms TNF signaling pathway. Endothelial proliferation and angiogenesis can also be affected by the *DPP4* siRNA treatment, as indicated by terms such as positive regulation of endothelial cell proliferation (GO:0001938), regulation of endothelial cell proliferation (GO:0001936), endothelial cell proliferation (GO:0001935), and positive regulation of vasculature development (GO:1904018). Monolayer permeability can also be affected by *DPP4* siRNA treatment, as indicated by the terms actin filament bundle assembly (GO:0051017), actin filament bundle organization (GO:0061572), and focal adhesion (Table 5A,B). Although previous studies demonstrated that LPS challenge provokes inflammation, the enhancement of monolayer permeability, and regenerative processes in pulmonary vascular endothelial cells [2], transcriptome analysis of HLMVECs stimulated by LPS has not been performed under these conditions. Our results suggest that comprehensive mechanistic pathways are involved in mediating the effects of LPS. Therefore, these transcriptome analysis results are useful for further understanding and exploring the functional responses of HLMVECs during LPS-induced inflammation.

Based on the results of the transcriptome data indicating a relationship between inflammation and CD26/DPP4, we characterized the possible differences in the expression of TNFα, IL-6, IL-8, and ICAM-1 at gene and protein levels, which are related to neutrophil inflammation, a hallmark of acute lung injury. The raw data for transcriptome analysis are summarized in Table 1, Table 3 and Table 5. We observed wide variations in gene expression levels, which may have occurred in part because we employed four lots of HLMVECs to make the results more universal. Heterogeneity, in the responses of these primary cells, is expected and likely contributed to the wide variability in gene expression. *IL6*, *CXCL8*, and *ICAM1* were upregulated by LPS, whereas *TNF* remained unchanged after LPS stimulation (Table 4). ICAM1 trended toward downregulation at both the gene and protein levels regardless of LPS stimulation (Table 1 and Table 6, Figure 3 and Figure 5Ai), whereas IL-6 and IL-8 expression at gene and protein levels were unchanged (Table 1 and Table 6, Figure 3 and Figure 5Aii). We previously demonstrated that pharmacological CD26/DPP4 inhibition in cultured HLMVECs reduced ICAM1 and IL-6 levels [17]. Together, CD26/DPP4 inhibition can exert an anti-inflammatory effect by reducing ICAM levels in HLMVECs, whereas other mechanisms related to pharmacological CD26/DPP4 inhibition, but not reduced CD26/DPP4 expression, can exist to reduce IL-6 release.

Based on the results of transcriptome analysis, suggesting a potential role for CD26/DPP4 in the barrier function of the pulmonary endothelium, we evaluated the effects of CD26/DPP4 suppression on the monolayer permeability of HLMVECs after LPS. Under the baseline conditions, without LPS stimulation, no terms related to barrier function were identified after *DPP4* knockdown via siRNA. However, after LPS stimulation, several terms related to monolayer permeability were enriched, and the term “tight junction” was identified after CD26/DPP4 suppression using siRNA. Tight junctions, intercellular adherent junctions, and gap junctions in endothelial monolayers are major determinants of barrier function between the blood and interstitial spaces in the lung [2] [30] [31]. Therefore, we evaluated the effect of CD26/DPP4 suppression on monolayer permeability. Although our data are limited, the results revealed a trend toward enhanced permeability after LPS challenge, which was further augmented by *DPP4* knockdown, suggesting that CD26/DPP4 can be involved in regulating monolayer barrier permeability in HLMVECs. Regarding the role of CD26/DPP4 in the permeability of endothelial monolayers, we previously reported that high concentrations of the pharmacological CD26/DPP4 inhibitor sitagliptin enhanced the permeability of the HLMVEC monolayer. In fact, clinical case reports have described the development of angioedema in some patients receiving CD26/DPP4 inhibitors (anagliptin and sitagliptin) [32,33]. Our results can provide a mechanistic explanation for the development of angioedema in these patients. Therefore, caution may be required when considering the use of CD26/DPP4 inhibitors in patients who are at an increased risk of vascular permeability. In contrast, it has been reported that pharmacological CD26/DPP4 inhibition by diprotin A helps maintain the VE-cadherin and adherens junctions of HUVECs under hypoxia by suppressing β-catenin cleavage [34]. Taken together, the role of CD26/DPP4 in regulating endothelial barrier function can be affected by the environment and/or types of endothelial cells and/or CD26/DPP4 inhibitors; however, further research is warranted to explore these details.

Transcriptome analysis suggested that CD26/DPP4 expression can be involved in the regenerative processes of the pulmonary endothelium. Based on these results, we evaluated the possible differences in related cell functions, such as proliferation, migration, and lumen formation. The suppression of CD26/DPP4 by siRNA promoted the cell proliferative capacity, but no changes in the migratory ability or suppression of tube formation were observed. Previous studies suggested that endothelial regeneration is required for the recovery from ARDS [22]; therefore, CD26/DPP4 suppression can promote endothelial repair, leading to the recovery from ARDS. However, how the migration or angiogenesis functions are related to the recovery from the pathophysiology of ARDS is unclear and requires further analysis.

Our study had several limitations. First, the details of localization and characterization of CD26/DPP4 in HLMVECs remain to be clarified, although CD26/DPP4 is known to be expressed on the surface of various cell types in the lung, and its expression levels on the cell surface of HLMVECs were suppressed via siRNA using flow cytometry analysis, in the present study (Figure 1A,B). Second, the effects of CD26/DPP4 overexpression in HLMVECs were not evaluated. Third, whether CD26/DPP4 suppression affects HLMVECs stimulated by LPS at different time points should be examined. Fourth, additional assays of monolayer permeability can be useful to better characterize the effects of CD26/DPP4 expression on the HLMVEC barrier function. Fifth, our results were obtained in vitro, and additional in vivo studies are needed to evaluate the effects of *DPP4* knockdown in an animal model of lung injury. Finally, and importantly, the effectiveness of CD26/DPP4 inhibition in patients with ARDS must be determined. Further research is ongoing to better understand these issues.

## 5. Conclusions

CD26/DPP4 expression can affect multiple key functions in pulmonary vascular endothelial cells, including inflammatory responses, monolayer permeability, and regenerative processes. These functions are all closely related to ARDS pathophysiology and the repair processes following lung injury. Thus, CD26/DPP4 is a potential therapeutic target for lung diseases that involve the dysfunction of pulmonary microvascular endothelial cells, such as ARDS.

## Figures and Tables

**Table 1:** Gene expression levels of ARDS-relevant factors (NC/PBS vs. *DPP4* siRNA/PBS). Normalized gene signals of each sample are shown in the table.

Gene	*p*-Value	FC	NC/PBS1	NC/PBS2	NC/PBS3	NC/PBS4	si/PBS1	si/PBS2	si/PBS3	si/PBS4
*DPP4*	0.052777	0.108789	3.6906	5.425	1.6427	−0.53626	−0.3191	−0.90569	−1.4262	0.071325
*ICAM1*	0.098061	0.391802	5.1235	4.9168	6.332	7.7584	4.3928	4.2642	5.2329	4.8335
*TNF*	0.424103	0.779134	−0.52021	−1.0127	0.49851	−0.20617	−1.3694	−0.31391	−0.15315	−0.84435
*IL6*	0.631241	1.251534	−0.81298	−0.57505	0.90575	0.064808	−1.078	0.67928	−0.0339	1.3099
*CXCL8*	0.181945	0.597338	5.0691	4.3318	5.2515	6.0029	5.0058	3.4162	4.458	4.8018

FC: fold change, si: *DPP4* siRNA, and NC: negative control siRNA.

**Table 2:** Enrichment analysis of transcriptomic data (*DPP4* siRNA/PBS vs. negative control/PBS). (A) Gene ontology (biological process): *DPP4* siRNA/PBS vs. negative control/PBS. (B) KEGG pathway: *DPP4* siRNA/PBS vs. negative control/PBS.

**(A)**	
**Term (Gene Ontology: Biological Process)**	***p*-Value**
positive regulation of endothelial cell proliferation (GO:0001938)	0.004012
regulation of endothelial cell proliferation (GO:0001936)	0.004224
endothelial cell proliferation (GO:0001935)	0.005302
vascular endothelial growth factor receptor signaling pathway (GO:0048010)	0.005728
positive regulation of vasculature development (GO:1904018)	0.008695
angiogenesis involved in wound healing (GO:0060055)	0.008703
vascular endothelial growth factor receptor-2 signaling pathway (GO:0036324)	0.015278
vascular endothelial growth factor signaling pathway (GO:0038084)	0.017529
positive regulation of angiogenesis (GO:0045766)	0.020147
**(B)**	
**Term (KEGG Pathway)**	***p*** **-Value**
MAPK signaling pathway	0.005904
Cell cycle	0.021388
TGF-beta signaling pathway	0.022962
TNF signaling pathway	0.02639
NF-kappa B signaling pathway	0.040451
VEGF signaling pathway	0.045165

**Table 3:** Enrichment analysis of transcriptomic data after LPS (NC/PBS vs. NC/LPS). KEGG pathway: NC/PBS vs. NC/LPS.

**Term (KEGG Pathway) with Upregulated Genes**	***p*-Value**
TNF signaling	<0.001
NF-kappa B signaling	<0.001
Chemokine signaling	<0.001
Toll-like receptor signaling	<0.001
JAK-STAT signaling	<0.001
PI3K-Akt signaling	0.0075459
MAPK signaling	0.0155037
**Term (KEGG Pathway) with Downregulated Genes**	***p*-Value**
Tight junction	0.021481

**Table 4:** Gene expression levels of ARDS-relevant factors after LPS (NC/PBS vs. NC/LPS). Normalized gene signals of each sample are shown in the table.

Gene	*p*-Value	FC	NC/PBS1	NC/PBS2	NC/PBS3	NC/PBS4	NC/LPS1	NC/LPS2	NC/LPS3	NC/LPS4
*DPP4*	0.711263	1.528313	3.6906	5.425	1.6427	−0.53626	3.5973	5.5607	1.7018	1.81
*ICAM1*	0.024742	4.432767	5.1235	4.9168	6.332	7.7584	7.953	7.791	7.8916	9.088
*TNF*	0.564979	0.790129	−0.52021	−1.0127	0.49851	−0.20617	0.34936	−1.5216	−1.3344	−0.093255
*IL6*	0.006241	4.722449	−0.81298	−0.57505	0.90575	0.064808	2.8088	2.0826	2.5762	1.073
*CXCL8*	0.000116	13.76836	5.0691	4.3318	5.2515	6.0029	9.6278	8.9046	8.3929	8.8631

**Table 5:** Enrichment analysis of transcriptomic data after *DPP4* siRNA and LPS (*DPP4* siRNA/LPS vs. negative control/LPS). (**A**) Gene ontology (biological process): *DPP4* siRNA/LPS vs. negative control/LPS. (**B**) KEGG pathway: *DPP4* siRNA/ LPS vs. negative control/ LPS.

**(A)**	
**Term (Gene Ontology: Biological Process)**	***p*-Value**
actin filament bundle assembly (GO:0051017)	0.001939
actin filament bundle organization (GO:0061572)	0.001939
positive regulation of endothelial cell proliferation (GO:0001938)	0.004012
regulation of endothelial cell proliferation (GO:0001936)	0.004224
endothelial cell proliferation (GO:0001935)	0.005302
vascular endothelial growth factor receptor signaling pathway (GO:0048010)	0.005728
positive regulation of vasculature development (GO:1904018)	0.008695
angiogenesis involved in wound healing (GO:0060055)	0.008703
regulation of actin filament depolymerization (GO:0030834)	0.011257
vascular endothelial growth factor receptor-2 signaling pathway (GO:0036324)	0.015278
vascular endothelial growth factor signaling pathway (GO:0038084)	0.017529
positive regulation of angiogenesis (GO:0045766)	0.020147
positive regulation of endothelial cell chemotaxis (GO:2001028)	0.02126
blood vessel endothelial cell proliferation involved in sprouting angiogenesis (GO:0002043)	0.022302
wound healing (GO:0042060)	0.023613
positive regulation of endothelial cell migration (GO:0010595)	0.023878
negative regulation of cell-substrate junction organization (GO:0150118)	0.02539
regulation of actin filament-based process (GO:0032970)	0.028945
**(B)**	
**Term (KEGG Pathway)**	***p*-Value**
TNF signaling pathway	0.006004
VEGF signaling pathway	0.032475
Focal adhesion	0.036127

**Table 6:** Gene expression levels of ARDS-relevant factors after *DPP4* siRNA and LPS (NC/LPS vs. *DPP4* siRNA/LPS). Normalized gene signals of each sample are shown in the table.

Gene	*p*-Value	FC	NC/LPS1	NC/LPS2	NC/LPS3	NC/LPS4	si/LPS1	si/LPS2	si/LPS3	si/LPS4
*DPP4*	0.009349	0.089367	3.5973	5.5607	1.7018	1.81	0.002409	−0.053284	−0.4756	−0.74028
*ICAM1*	0.173762	0.690418	7.953	7.791	7.8916	9.088	7.3559	7.4082	7.7555	8.0661
*TNF*	0.662287	1.189233	0.34936	−1.5216	−1.3344	−0.093255	−0.92489	0.095058	0.11528	−0.88522
*CXCL8*	0.434415	0.817062	9.6278	8.9046	8.3929	8.8631	9.3594	8.3247	8.4618	8.4767
*IL6*	0.580922	0.846619	2.8088	2.0826	2.5762	1.073	1.6601	2.2974	1.7002	1.9221

## Data Availability

The data presented in this study are available upon request from the corresponding authors. Datasets analyzed or generated during the study were archived in the GEO database: GSE190405.

## Supplementary Materials

The following materials are available online at https://www.mdpi.com/article/10.3390/cells10123508/s1. Figure S1: A representative figure showing the way of quantification of tube formation assay. The number of branch nodes (presented as white asterisks) was counted and the lengths of all the tubes (presented as white arrow heads) were measured. The numbers of branch nodes and lengths were respectively summed per one field of a microscope image of each well. Figure S2: Representative images regarding the wound healing assay.
