# Supplementary material for: Functional Roles for CD26/DPP4 in Mediating Inflammatory Responses of Pulmonary Vascular Endothelial Cells
Source: Cells. 2021 Dec 11;10(12):3508. doi: 10.3390/cells10123508 (PMC8700481; doi:10.3390/cells10123508)

Supplementary Figure S1

Representative image regarding quantification of tube formation assay

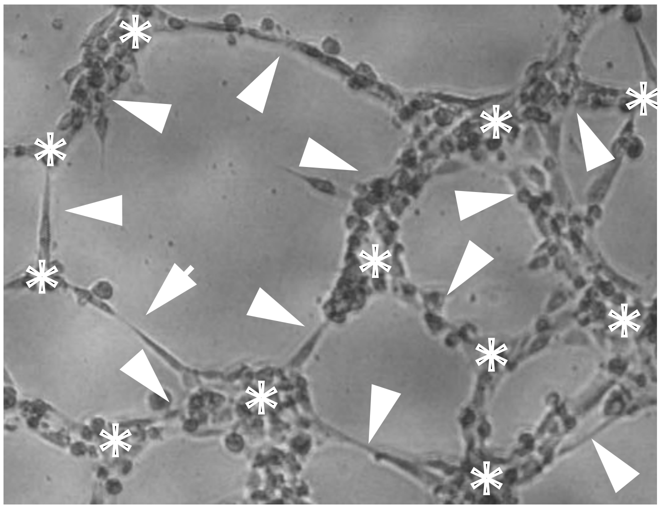

Supplementary Figure S2

Representative images regarding wound healing assay

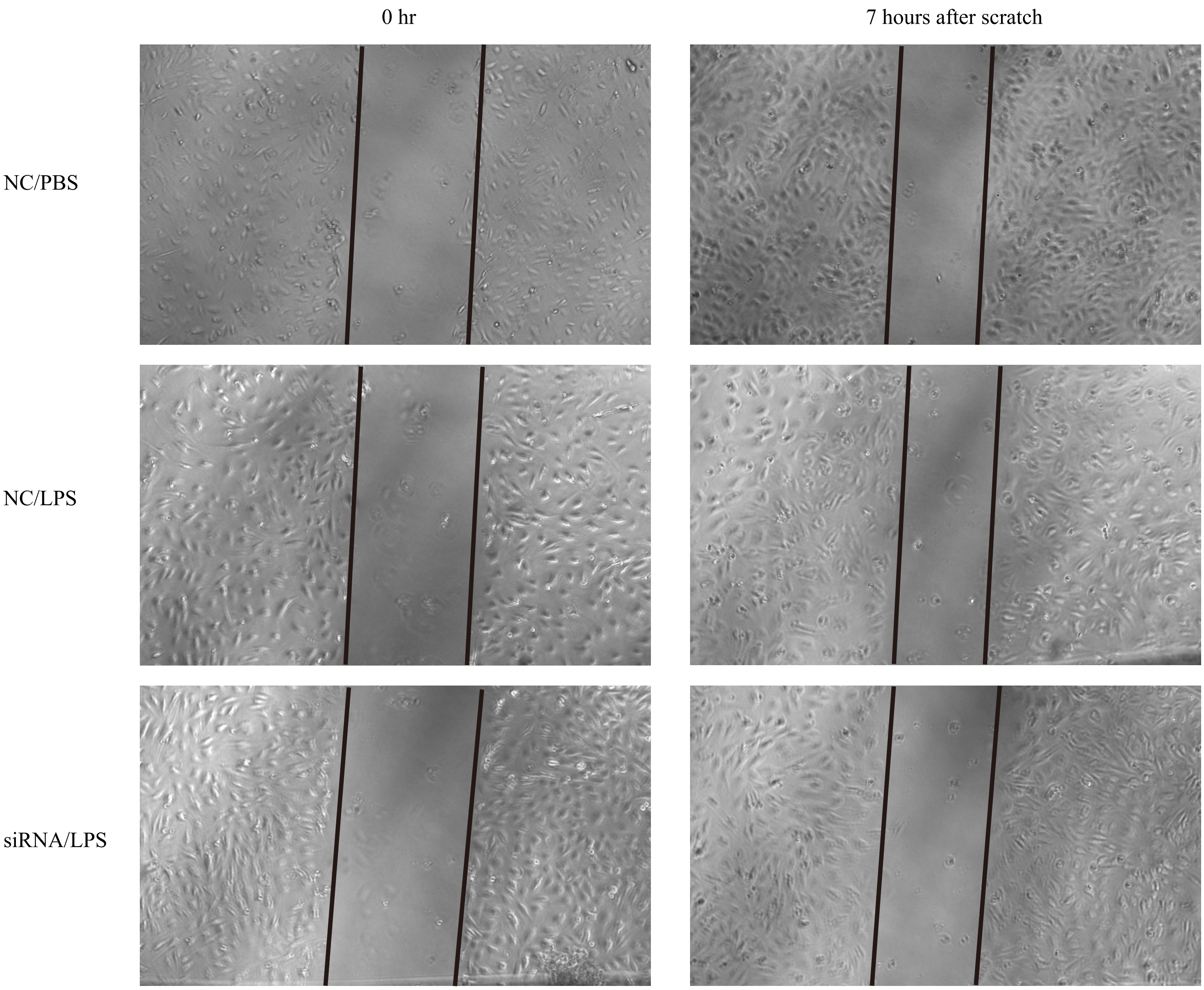

Supplement: Supplementary file 1 [file cells-10-03508-s001.zip › cells-1485675-supplementary.pdf]
